# Physical Aspects of Healthy Aging: Assessments of Three Measures of Balance for Studies in Middle-Aged and Older Adults

**DOI:** 10.1155/2010/849761

**Published:** 2011-03-03

**Authors:** Clementina D. Ceria-Ulep, John Grove, Randi Chen, Kamal H. Masaki, Beatriz L. Rodriguez, Tim A. Donlon, Jack Guralnik, Bradley J. Willcox, D. Craig Willcox, Claudio Nigg, J. David Curb

**Affiliations:** ^1^Department of Nursing, School of Nursing and Dental Hygiene, University of Hawaii at Manoa, 2528 McCarthy Mall, Honolulu, HI 96822, USA; ^2^Department of Public Health Sciences, John A. Burns School of Medicine, University of Hawaii, Honolulu, HI 96822, USA; ^3^Pacific Health Research Institute, Honolulu, HI 96813, USA; ^4^Department of Geriatric Medicine, John A. Burns School of Medicine, University of Hawaii, Honolulu, HI 96822, USA; ^5^Department of Cell and Molecular Biology, John A. Burns School of Medicine, University of Hawaii, Honolulu, HI 96822, USA; ^6^Department of Pathology, John A. Burns School of Medicine, University of Hawaii, Honolulu, HI 96822, USA; ^7^Laboratory of Epidemiology, Demography and Biometry, National Institute on Aging, National Institutes of Health, Bethesda, MD 20892, USA; ^8^Okinawa International University, Ginowan City, Okinawa 901-2701, Japan

## Abstract

*Objectives*. To investigate the reliability and correlations with age of the balance components of the EPESE, NHANES, and the Good Balance Platform System (GBPS) in a normal population of adults. *Design*. Cross-sectional. *Setting*. Urban Medical Center in the Pacific. *Participants*. A random sample of 203 healthy offspring of Honolulu Heart Program participants, ages 38–71. *Measurements*. Subjects were examined twice at visits one week apart using the balance components of the EPESE, NHANES, and the good balance system tests. *Results*. The EPESE and NHANES batteries of tests were not sufficiently challenging to allow successful discrimination among subjects in good health, even older subjects. The GBPS allowed objective quantitative measurements, but the test-retest correlations generally were not high. The GBPS variables correlated with age only when subjects stood on a foam pad; they also were correlated with anthropometric variables. *Conclusion*. Both EPESE and NHANES balance tests were too easy for healthy subjects. The GBPS had generally low reliability coefficients except for the most difficult testing condition (foam pad, eyes closed). Both height and body fat were associated with GBPS scores, necessitating adjusting for these variables if using balance as a predictor of future health.

## 1. Introduction

Assessment of balance is important, especially in the elderly, since balance affects the ability of the individual to be mobile and functionally independent [1]. The term “balance” encompasses several different types of control mechanisms for stability including vestibular function [2], visual cues [3], the proprioceptive system [3, 4], and muscle control [4]. 

Balance has been measured in different ways including the use of force platforms [5] with both eyes either open or closed [6, 7], using the Berg Balance Scale [8], the Romberg stand, semi-tandem, full tandem, and side-by-side/parallel leg stands [9, 10]. Balance performance has been assessed dually with cognitive tasks [7, 11], with postural disturbance [12], and after a stroke rehabilitation [13]. A complicating factor is that some tests of balance might require simple muscular strength in addition to balance ability [14].

The quantification of balance, including the examination of sway [15–17], has been extensively studied among normal and subjects with some balance abnormality [18, 19] and on various age groups [20]. Psychometric properties have been established for various balance measures utilizing older adults including but are not limited to Berg Balance Scale and Multidirectional Reach Test [21]; side-step test [22]; Fullerton Advanced Balance Scale [23]; Late-Life Function and Disability Instrument [24]; Dynamic Gait Index [25], and Activities-specific Balance Confidence Scale and the Survey of Activities and Fear of Falling in the Elderly [26]. These studies have helped define and improve the geriatric definition and utility of balance but have not addressed its potential utility as a predictor of future functional capacity in healthy adults. The purpose of the present study was to test the reliability of specific measures of balance to be included in an enhanced battery of measures of functional ability that would allow better discrimination among a random sample of individuals. Like grip strength [27], balance might be a predictor of future health. For research purposes, it is important to appraise methods of measuring balance to obtain a set of informative tools. This is rather different from using physical performance to detect current disease, since we hope to distinguish among people at the upper end of performance; at present, tools to assess balance are geared towards those with balance weakness or deficit. 

The focus of this paper is to evaluate three measures of balance: the balance components of the EPESE [28] and NHANES [29] tests and the Good Balance Platform System [30]. The authors consulted with an internationally recognized panel of experts who recommended these commonly used measures possessing elements that test a broad range of functional levels. See Curb et al. [31] for further details. The reliability, correlations of these different tests with age, and correlations between the tests will be presented. To the best of our knowledge, this is the first published report of a reliability study comparing these three methods of measuring balance. Establishing the reliability and validity of balance measures is important to assess their suitability for use in clinical practice and research.

## 2. Methods

### 2.1. Study Design

The sample consisted of noninstitutionalized individuals, Japanese Americans who were drawn from lists of offspring of the Honolulu Heart Program participants, an epidemiological long-term cohort of 8,006 Japanese-American men in Hawaii [32]. These participants were randomly selected into two age groups: 35–55 and 56–71 years old. The two age groups provided a range in age and diversity in functional ability. Although the study required two examinations, two hundred ten agreed to participate for a recruitment rate of 50%. There were 105 per group stratified equally by sex. However, only 203 participants completed the two examinations—three did not return, two cancelled, and two did not have blood drawn. The two examinations were approximately a week apart. The first examination included a questionnaire on demographics, family and medical history, lifestyle, anthropometry (hip, seated mid-calf and waist circumference, maximum sagittal width, subscapular, and triceps skinfold), physical activity and function measures, and other physical measures such as heart rate, blood pressure, blood sample, and cognitive assessment. The second visit included all the measures except for the questionnaire, anthropometric measures, and blood draw. The first and second examination, took 2.5 to 3 hours and 1.5 to 2 hours, respectively. See Curb et al. [31] for further details on examinations and measures. Approval to conduct the study was given by the institutional review committee of Kuakini Medical Center where the study was performed, and informed consent was obtained from all participants.

### 2.2. Measurement of Variables

#### 2.2.1. Established Populations for Epidemiologic Studies of the Elderly (EPESE) Battery of Tests

The EPESE battery includes semi-tandem, side-by-side, and fulltandem stands. This graded series of tests measures static balance while standing still for 10 and 30 seconds. For the semi-tandem test, the participant is instructed to stand with the side of the heel of one foot touching the big toe of the other foot for 10 seconds. Participants who cannot perform this proceed to the side-by-side stand. With the side-by-side stand, the participant is instructed to stand with his/her feet together, side-by-side for 10 seconds. If the semi-tandem stand test is passed, the participant proceeds to the fulltandem stand, with the heel of one foot in front of and touching the toes of the other foot for 30 seconds. 

#### 2.2.2. NHANES Balance Test

The Romberg Test of Standing Balance on Firm and Compliant Support Surfaces from the ongoing National Health and Nutrition Examination Surveys (NHANESs) of the National Center of Health Statistics required the participant to stand under four different conditions, on a hard stationary surface with eyes open/closed for 15 seconds and on a foam pad with eyes open/closed for 30 seconds each. The EPESE and NHANES measures were scored as qualitative pass/fail assessments of balance.

#### 2.2.3. Good Balance Platform System

For the third set of tests, we incorporated the Romberg Test's four conditions into the *Good Balance Platform System* (GBPS) from Finland. The *GBPS *converts shifts in weight to digital data to obtain a quantitative assessment of maintenance of balance. The components of the system include a force platform and a handrail that wraps around the front and sides for safety. The *GBPS* records several functions of the amount and speed of the subject's mediolateral (ML) and anterior-posterior (AP) sway over a specified duration of time (our exams used 15 and 30 seconds). Table 1 describes the balance platform variables associated with the displacements.

Subjects stood for 30 seconds per test using the same four conditions as the NHANES test (hard surface with eyes open, then closed, followed by a foam surface with eyes open, then closed) and with the same safety precautions. For the first two conditions, the participants stood in the center of the triangular platform with their bare feet about a foot apart, with their hands together in front, right hand cupping the left, and the arms kept straight. For the last two conditions on a foam pad, the participant was instructed to stand with arms folded across the waist, holding the elbows with the hand (NHANES arm position). The use of the foam pad was adapted from the NHANES measures and was not part of the normal protocol recommended for the balance platform. Apparently, this is the first report of the use of the foam pad in combination with a computer-linked balance platform.

### 2.3. Analysis

For tests which were graded as pass/fail, such as the NHANES battery of tests, Fisher's exact test was used to test for association between repeated tests. Pearson product-moment correlations were used to estimate test-retest correlations; these estimate the intraclass correlation coefficients (the usual estimate of the reliability coefficients) but allow for a shift in means across visits in case there was some degree of learning experience. The effect of gender and physical characteristics on quantitative outcome variables was appraised using multiple linear regression models.

## 3. Results

A total of 203 subjects completed both visits, 87 aged 38–55 and 116 aged 56–71. There were 97 females and 106 males. The sample did not include the extreme elderly as the mean age was only 58. Generally, the participants were in reasonably good health, with no reported history of heart attack, stroke, or cancer. However, 46% were on medication for hypertension, 11% were being treated for diabetes, and 43% and 14% met body max index (BMI) WHO [33] criteria for overweight and obese, respectively. Refer to Tables 2 and 3 for further details.

Tests for which all participants pass (or all fail) do not have defined reliability coefficients because there is no variability across subjects (the calculation of the correlation coefficient would have division by zero). For the EPESE battery of tests of balance, all participants could perform the semi-tandem stand at both visits, while only 6.4% and 2.5% could not perform the fulltandem stand at visits 1, and 2, respectively. Since all of the participants could perform the semi-tandem stand, they did not have to do the easier side-by-side stand.

The NHANES set had four tests of balance, standing on a flat surface with eyes either open or closed, and standing on a foam pad with eyes either open or closed. For the standard, eyes open condition, all participants passed the test while for the standard, eyes closed condition, only one participant could not pass it at one exam only. For the foam pad, eyes open test, less than one percent could not pass the test, and only one person failed both exams. The foam pad, eyes closed test was somewhat more informative, with 9% and 6% of the participants at visits 1 and 2, respectively, unable to perform it. However, its reliability correlation was only 0.26 (which was still significantly greater than zero, *P* < .001 by Fisher's exact test). Since the majority of the participants passed the EPESE and NHANES tests with the exception of the single leg stand, we did not do any further analysis such as adjusting for height and weight. Table 4 is a summary of the NHANES and EPESE balance tests.

Estimated reliability coefficients for the numerous *Good Balance Platform System* variables ranged from 0.22 to 0.73. While some variables such as “correlation” and “main axis” had low-reliability coefficients under all four testing conditions, the variables “mean X -speed,” “mean Y-speed,” and “velocity moment” had reasonably high-reliability coefficients under the most difficult testing condition (foam pad, eyes closed). To improve the reliability coefficient of these variables, we examined scatterplots and evaluated the following:

1. deletion of outliers (for two participants, their values for some variables at the second exam were wildly discrepant with their first exam values);
2. transformation by taking the logarithm;
3. deletion of outliers and log transformation.

**Figure 1:**
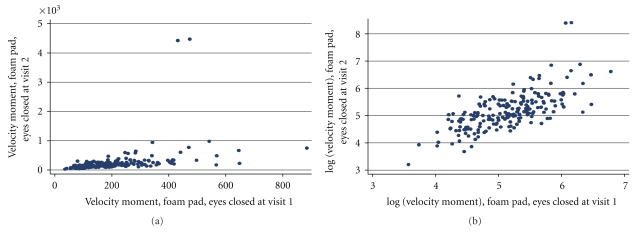
Velocity moment, foam eyes closed, before (a) and after (b) log transformation.

Table 5 summarizes the reliability coefficients in the original scale and after taking logs (some variables had a constant added to them to make the lowest value equal to 1 before log transformation). The reliability coefficients of five out of ten variables increased meaningfully after transformation, while the reliability of a few decreased by log transformation. Remarkably, removing two extreme outliers resulted in less improvement than simple log transformation (results not shown). Figures 1(a) and 1(b) display plots of the variable “velocity moment” (foam pad, eyes closed) before and after log transformation.

Apart from “mean Y-speed” (average speed moving front-to-back), Good Balance Platform System variables were correlated with age only when subjects stood on a foam pad (see values in parenthesis in Table 5). Under easier conditions (i.e., standard, eyes open and closed), the balance variables had low correlations with age, in keeping with their low reliability coefficients.

Further analysis in evaluating means and standard deviations showed that as test condition difficulty increased, so too did the variables' means and standard deviations, with foam, eyes closed having the most substantial impact. For example, for “mean Y-speed,” the means (and standard deviations) are 6.2 (2.0), 8.9 (3.0), 12.6 (3.6), and 26.0 (8.3) for the four conditions, standard eyes open and closed, foam pad eyes open and closed, respectively. The increase in the standard deviation suggests that the effect of increasing the difficulty is not the same for everyone. When the values are log transformed, the standard deviations are nearly constant and range from about 0.3 to 0.35; this constancy of variance of log-transformed values improves their statistical properties if used as dependent variables. 

The four variables with the highest reliability coefficients, “velocity moment,” “mean X-speed,” “mean Y-speed,” and “length of side of square” were regressed on age and gender using the easiest and most difficult conditions, standard, eyes open and foam pad, eyes closed. The effect of age increased with difficulty of test condition. Women had better (lower) average balance scores than men, the advantage being greater under the more difficult testing condition.

We investigated the relationships between the four most reliably measured *GBPS* variables and (1) measures of body fat (waist circumference, sagittal diameter, mid-calf, triceps, and subscapular skinfold thicknesses), (2) distance walked in six minutes, and (3) failure to pass the *NHANES* full tandem stand on a foam surface, eyes closed. To improve the reliability of the variables, the averages of exam 1 and exam 2 *GBPS* variables, the distance walked in six minutes, and the total number of “failures” of the *NHANES* test were used. We included the 6-minute walk in the analysis because of its high reliability (.90) in this and Harada et al.'s [34] study, and its relationship to balance [34]. All variables were adjusted for age and gender. 

(1) All five measurements of body fat were negatively correlated with all *GBPS* variables, with correlations being significant for three to four *GBPS* variables per body fat variable. These correlations could be substantial; for instance, the correlation between subscapular skinfold thickness and log “mean Y speed” was *r* = −0.37 (*P* < .0001). This means fatter people had better balance or less movement on the platform. (2) The distance walked in six-minutes was positively correlated with log “mean Y speed” (*P* = .023) and log “mean X speed” (*P* = .006) and of borderline significance with log “velocity moment” (*P* = .06); that is, greater movement on the balance platform was correlated with faster walking. The correlation between the six-minute walk score and balance platform values diminished after adjusting for height, with only log of “mean X speed” remaining significant. The difference between genders in means of six minute walk scores and balance platform scores also disappeared after adjusting for height. (3) The total number of failures for the *NHANES* full tandem stand on a foam surface, eyes closed test was highly significantly correlated with all four *GBPS* variables, even though a single *NHANES* test had low reliability; using the average of the values over two exams improved the results. The correlations were 0.37, 0.23, 0.26, and 0.39 for log “velocity moment,” log “mean X speed,” log “mean Y speed,” and log “length of square,” respectively. The correlations between the sum of failures for NHANES foam pad, eyes closed with the balance platform scores remained high after adjusting for age, gender, height, and body mass index (BMI).

## 4. Discussion

The purpose of this study was to obtain tests of balance with good reliability to distinguish among people with at least normal functional abilities. None of the various measurements of balance had outstanding reliability coefficients in our sample of healthy men and women. The least discriminatory test was the EPESE battery of tests, since nearly all participants could perform the tests successfully, and those who failed to pass one test usually could succeed during the other examination. However, in another study [35] of adults aged 55–70 years old, semi-tandem and tandem stands had good reliability. For the NHANES set, most of the participants could pass all tests except for the foam pad, eyes closed testing condition, which averaged 8% failure but had a low-reliability coefficient. Thus, these tests do not appear to have been sufficiently challenging for subjects in good health, even among our older subjects, to allow discrimination between levels of function.

The *Good Balance Platform System* seemed promising since it allowed a quantitative score rather than a qualitative pass/fail result. Even so, the reliability coefficients were disappointing when subjects stood on a hard surface, whether or not their eyes were closed. Only one variable (“mean Y speed”) had a reliability coefficient as high as 0.7. Standing on a foam pad increased the difficulty, particularly when the subjects had their eyes closed, which increased the test-retest correlation coefficients for half of the variables. Log transformation of the scores reduced heteroscedasticity and skewness and increased the reliability coefficients of several balance variables, but only three of the reliability coefficients were greater than 0.7 even for the foam pad, eyes closed test, the most difficult condition. 

Women generally had better balance scores on the balance platform than men, which is consistent with Røgind et al.'s [36] findings but contrary to the findings of Wolfson et al. [37]. We found that this difference disappeared after adjusting for height. For four balance platform variables with the highest reliability coefficients, increased movement on the balance platform was associated with good performance with the six-minute walk. These associations also diminished after adjusting for subject's height, with only one variable remaining significantly correlated. In contrast, the correlations between the sum of failures for NHANES foam pad, eyes closed with balance platform scores remained high after adjusting for age, gender, height, and BMI. Surprisingly, individuals with increased waist and mid-calf circumference and skinfold measurement had better balance, contrary to other findings [38, 39]. It should be pointed out that 14% of the subjects in the present study were obese. The correlations of balance scores with body fat means that investigators might misinterpret the health implications of GBPS balance score: we found that diastolic blood pressure had significant negative correlations with several GBPS variables, but that these correlations disappeared after adjusting for BMI (results not shown).

As test condition difficulty increased across the four conditions, so too did the mean and standard deviations, with foam pad, eyes closed being impacted the most. The increase in standard deviation apparently means that the effect of increasing test difficulty is not the same for all subjects. Four variables (“velocity moment,” “mean X-speed,” “mean Y-speed,” and “length of side of square”) with the highest test-retest reliability coefficients were regressed on age and gender using the easiest and most difficult conditions—standard, eyes open and foam, eyes closed. We found that the effect of age increased with difficulty of test condition and that women had better (lower) balance scores than men, the advantage being greater under the more difficult testing condition. Our results are consistent with other studies that reported low-reliability coefficients for computerized balance platforms unless subjects faced additional challenges [7, 40, 41]. Some of these challenges required specialized equipment [41]. We found that the simple addition of a foam pad and log transformation frequently increased the reliability coefficient substantially. 

 Since difficulty in maintaining balance varied across the testing conditions, taking the difference between the easiest and most difficult conditions might have generated meaningful balance variables. The difference in scores generally increased with age, reflecting the greater difficulty the elderly experienced with the foam pad. However, the difference in scores generally had rather low reliability coefficients, with the best (for difference in log “mean Y speed”) being only 0.66. Remarkably, the *GBPS* variable “correlation” had a test-retest correlation for the difference in scores of 0.65 although the reliability coefficients for each separate measurement was low. The meaning of a severely worsened balance score caused by using a more difficult testing condition is still largely unexplored at this time, but a difference in scores might be a useful complement to the more usual measurements. 

Investigators might want to consider measuring participants' balance variables more than once to increase the reliability of the value. The reliability coefficient of values which are the means of m independent measurements per subject can be expressed as *ρ*_m_ = (1+ [1−*ρ*]/m)^−1^, where *ρ* is the reliability coefficient of a single measurement. If the reliability coefficient of a measurement were, say 0.70, then using the average of two independent measurements per subject would increase the reliability coefficient to 0.82. While it probably would be best to have balance measurements measured at least a few days apart to insure that they are independent, one might improve the reliability of the variables a fair amount simply by having subjects repeat the balance platform test a second and third time during the same examination.

A limitation to this study needs to be considered. Data reported in this study may not be applicable to other ethnic groups since the subjects were of unmixed Japanese descent living in Hawaii.

## 5. Conclusions

The EPESE and NHANES tests, which are scored as pass/fail, were too easy for healthy subjects and did not allow differentiating among people. The Good Balance Platform System (GBPS) variables, which are quantitative, had rather low-reliability coefficients except under the most difficult testing condition (standing on foam pad with eyes closed) and generally were not correlated with age except when subjects stood on a foam pad; five out of ten of the variables had their reliability coefficients improved appreciably by using a log transformation of the scores. Taking the average of multiple balance platform measurements would improve the reliability even more. The GBPS variables were positively correlated with height and negatively correlated with measures of body fatness; for research on the value of balance as a predictor of future health, adjustment for height and body fat or relative weight should be made.

## Figures and Tables

**Table 1:** Balance platform main variables (×4 tests).

Variable name	Description
Mediolateral (ML) sway	Distance which contains 90% of lateral displacement of center of forces
Anterior-posterior (AP) sway	Distance which contains 90% of anterior-posterior displacement of center of forces
Length of side of square	Length of the smallest square containing 90% of path of center of forces
Mean X speed	Average speed of lateral movement of center of forces
Mean Y speed	Average speed of anteroposterior movement of center of forces
Velocity moment	Average horizontal area covered by movement of center of forces per second
Correlation	Correlation between lateral and antero-posterior movement of center of forces
Direction of main axis	Angle of direction of average movement of center of forces
	Right = 0 degrees
	Forward = 90 degrees
	Left = 180 degrees
	Backward = 270 degrees
Mean X value	Average value of center point on lateral axis (left is negative)
Mean Y value	Center point on anterior-posterior axis—leaning to the front/back

**Table 2:** Age, gender, medical history and body max index (BMI) (*N* = 203).

Age group (years)	% of sample
38–44	5.91
45–54	33.50
55–64	33.50
65–71	27.09

Gender	
Female	47.57
Male	52.43

Medical history	
Cancer	0.00
Heart attack/myocardial infarction (MI)	0.00
Stroke	0.00
On diabetes medication	11.33
On hypertension medication	46.00

*BMI categories	
<18.5 (underweight)	1.97
18.5–24.99 (normal range)	40.39
25.00–29.99 (preobese)	43.35
>30 (obese)	14.29

*Note: According to World Health Organization (WHO) [33].

**Table 3:** Sample: Age and Anthropometric Measures (*N* = 203).

Variable	Mean	Minimum	Maximum	Standard deviation
Age	57.95	38.00	71.00	8.35
BMI	26.08	17.26	48.96	4.70
Height (cm)	161.48	137.90	182.00	8.72
Weight (kg)	68.32	38.00	119.60	14.63
Hip (cm)	97.09	74.00	142.00	8.48
Waist (cm)	89.21	62.00	140.00	11.94
Mid-calf circumference (cm)	36.64	28.70	52.00	3.57
Triceps skinfold (cm)	18.47	4.30	60.70	9.29
Subscapular skinfold (cm)	22.58	7.00	51.00	8.27
Maximum sagittal width (cm)	21.04	14.50	30.80	2.94

**Table 4:** NHANES and EPESE balance test results at visit 1 and visit 2.

Movement	Pass visit 1	Pass visit 1	Fail visit 1	Fail visit 1
	Pass visit 2	Fail visit 2	Pass visit 2	Fail visit 2
NHANES				
Standard, eyes open	203	0	0	0
Standard, eyes close	202	1	0	0
Foam pad, eyes open*	201	0	1	1
Foam pad, Eyes close**	176	8	14	5
EPESE				
Semi-tandem	203	0	0	0
Full tandem	187	11	3	2

Test for association between visit 1 and visit 2 results: *P < .01; **P < .001.

**Table 5:** Balance platform—test-retest reliability *N* = 203.

Variable name	Standard, eyes open	Standard, eyes closed	Foam, eyes open	Foam, eyes closed
ML sway	0.36^‡^	0.38^‡^	0.29^‡^	0.34^‡^
	(0.08)	(0.04)	(0.28^†^)	(0.32^†^)
Log ML sway	0.38^‡^	0.46^‡^	0.32^‡^	0.56^‡^
AP sway	0.29^‡^	0.39^‡^	0.36^‡^	0.36^‡^
	(0.12)	(0.09)	(0.14)	(0.36^†^)
Log AP sway	0.33^‡^	0.46^‡^	0.37^‡^	0.55^‡^
Length of side of square	0.29^‡^	0.29^‡^	0.43^‡^	0.43^‡^
	(0.12)	(0.12)	(0.21**)	(0.21**)
Log length of side of square	0.33^‡^	0.47^‡^	0.45^‡^	0.61^‡^
Mean X speed	0.42^‡^	0.36^‡^	0.62^‡^	0.64
	(0.07)	(0.02)	(0.32^†^)	(0.24^†^)
Log mean X speed	0.46^‡^	0.53^‡^	0.65^‡^	0.71^‡^
Mean Y speed	0.70^‡^	0.68^‡^	0.73^‡^	0.72^‡^
	(0.34^†^)	(0.26^†^)	(0.37^†^)	(0.29^†^)
Log mean Y speed	0.69^‡^	0.69^‡^	0.72^‡^	0.76^‡^
Velocity moment	0.37^‡^	0.46^‡^	0.49^‡^	0.40^‡^
	(0.15*)	(0.07)	(0.34^†^)	(0.31^†^)
Log velocity moment	0.42^‡^	0.59^‡^	0.55^‡^	0.70^‡^
Correlation	0.15*	0.24^†^	0.21**	0.04
	(0.07)	(0.002)	(−0.07)	(−0.0006)
Log correlation	0.18*	0.23^†^	0.21**	0.03
Main axis	−0.10	0.16*	0.13	0.09
	(−0.07)	(0.03)	(0.01)	(−0.04)
Log main axis	0.02	0.16	0.13	0.06
Mean X value	0.48^‡^	0.34^‡^	0.31^‡^	0.31^‡^
	(0.04)	(0.02)	(0.005)	(−0.10)
Log mean X value	0.35^‡^	0.26^†^	0.20**	0.24^†^
Mean Y value	0.28^‡^	0.34^‡^	0.22^†^	0.25^†^
	(−0.06)	(−0.05)	(0.17*)	(0.21**)
Log mean Y value	0.23**	0.21**	0.23**	0.27^‡^

*P < .05; **P < .01; ^†^ *P* < .001; ^‡^ *P* < .0001.

The values in parenthesis are the age correlation for visit 1.
